# Intestinal Tuft Cells: Morphology, Function, and Implications for Human Health

**DOI:** 10.1146/annurev-physiol-042022-030310

**Published:** 2023-10-20

**Authors:** Jennifer B. Silverman, Paige N. Vega, Matthew J. Tyska, Ken S. Lau

**Affiliations:** Epithelial Biology Center and Department of Cell and Developmental Biology, Vanderbilt University School of Medicine, Nashville, Tennessee, USA

**Keywords:** tuft cells, cytoskeleton, chemosensation, inflammation, microbiome, differentiation

## Abstract

Tuft cells are a rare and morphologically distinct chemosensory cell type found throughout many organs, including the gastrointestinal tract. These cells were identified by their unique morphologies distinguished by large apical protrusions. Ultrastructural data have begun to describe the molecular underpinnings of their cytoskeletal features, and tuft cell–enriched cytoskeletal proteins have been identified, although the connection of tuft cell morphology to tuft cell functionality has not yet been established. Furthermore, tuft cells display variations in function and identity between and within tissues, leading to the delineation of distinct tuft cell populations. As a chemosensory cell type, they display receptors that are responsive to ligands specific for their environment. While many studies have demonstrated the tuft cell response to protists and helminths in the intestine, recent research has highlighted other roles of tuft cells as well as implicated tuft cells in other disease processes including inflammation, cancer, and viral infections. Here, we review the literature on the cytoskeletal structure of tuft cells. Additionally, we focus on new research discussing tuft cell lineage, ligand-receptor interactions, tuft cell tropism, and the role of tuft cells in intestinal disease. Finally, we discuss the implication of tuft cell-targeted therapies in human health and how the morphology of tuft cells may contribute to their functionality.

## INTRODUCTION

The gut barrier is maintained by a tightly packed monolayer of columnar epithelial cells; although the vast majority of these cells are solute transporting (e.g., enterocytes), this monolayer also includes specialized secretory cells within it including goblet, Paneth, and tuft cells (1). Goblet cells secrete mucins that build a mucous layer to reduce microbial contact with the epithelium, while Paneth cells secrete antimicrobials that diffuse into the mucous layer, further reducing microbial colonization (1, 2). In contrast, the full complexity of tuft cells’ functions in maintaining a healthy epithelium is still being uncovered.

Tuft cells were first characterized independently by two groups in 1956 (3, 4) and were given their name based on the morphological observations of the tuft-shaped apical protrusions that extended into the luminal space above that of their neighboring epithelial cells (5). Other distinctive tuft cell features include the large filamentous network composed of microtubules, actin, and intermediate filaments that stretch from the apical surface down to the perinuclear region of the cell interdigitating with the extensive tubulovesicular network (6, 7).

Tuft cells have been identified in multiple organs over the last several decades, including the thymus, urethra, stomach, intestine, biliary system, airways, and recently, in pancreatic precancerous lesions (6, 8–14). While existing studies allude to organ-specific functions, tuft cells are generally considered a specialized chemosensory cell type that elicits responses based on luminal or microenvironmental stimuli.

In the intestinal epithelium, tuft cells comprise about 0.4–2% of all epithelial cells under homeostatic conditions (15, 16). However, tuft cell proportions change dramatically in response to commensal microbes, pathogens, inflammation, and damage conditions (17–19), suggesting a fundamental role for this cell type in barrier function. Among the most understood tuft cell contributions in this context is the “weep and sweep” response that occurs in response to helminths via tuft cell release of interleukin (IL)-25 that stimulates subsequent release of IL-13 by type 2 innate lymphoid cells (ILC2s). Elevated levels of IL-13 act upon stem cells to increase specification of tuft and goblet cells, aiding in parasite clearance (20–22). Although tuft cell responses to helminths have been expertly reviewed (19, 23–31), it has recently become clear that tuft cells also play other direct and indirect roles in shaping the immune response to microbes, such as bacteria, protists, and viruses. In this review, we discuss the most recent studies that uncover these lesser-known roles of tuft cells. Moreover, we summarize knowledge about the morphology and subcellular structure of tuft cells, the ligand-receptor interactions that stimulate tuft cells, and the effector functions that tuft cells elicit in the complex intestinal environment. Finally, we discuss the various roles of intestinal tuft cells in disease, including inflammation, cancer, and infection.

## TUFT CELL MORPHOLOGY

**Figure 1:**
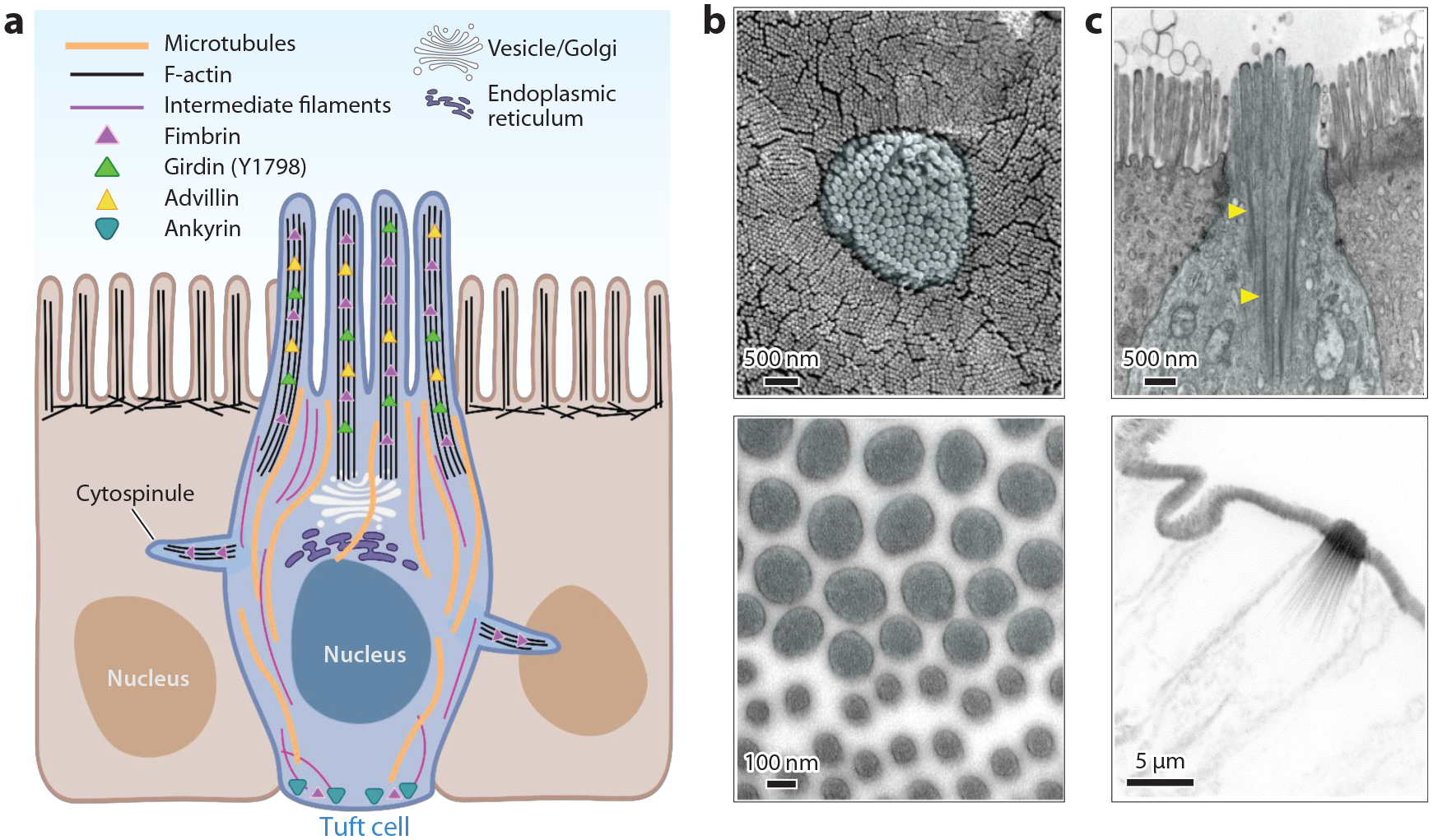
Tuft cell cytoskeletal morphology. (*a*) Graphic depicting major organelles and cytoskeletal components and their spatial organization within a tuft cell. Panel adapted from images created with BioRender.com. (*b*, *top*) En face scanning electron microscopy image demonstrating tuft cell apical protrusions next to neighboring microvilli. (*Bottom*) En face transmission electron microscopy image of tuft cell apical protrusions next to neighboring microvilli. (*c*, *top*) Lateral transmission electron microscopy image of tuft cell; yellow arrows point to core actin bundles. (*Bottom*) Phalloidin stain for F-actin.

Tuft cells are morphologically unique with distinctive cytoskeletal characteristics that act as identifiers of this cell type throughout the many different tissues and organs in which they are found. Generally, tuft cells are 17–20 μm tall with a narrow apical surface that widens to 7–13 μm at the nucleus (10) and may narrow again at the basal membrane, giving tuft cells a triangular (6) or bottle-shaped appearance (32). Most evident of their distinctive features is the tuft of large protrusions on their apical surface; in the context of the intestinal epithelium, these structures are supported by a unique actin cytoskeletal architecture and extend well past the microvilli that protrude from neighboring enterocytes (33) (Figure 1a).Since their initial characterization, many morphological and ultrastructural studies on tuft cells have been conducted in tissues across several species including humans, placental mammals, bullfrogs, and some species of fish (13,34–36).Our current understanding of the tuft cell cytoskeleton derives from conventional electron microscopy methods and immunostaining of tissue sections. While these methods have gained sophistication over time and have offered a basic framework for thinking about how tuft cells contribute to tissue physiology, the fundamental cell biology governing tuft cell function remains largely unexplored. Below, we review the limited knowledge on the structural/cytoskeletal underpinning of tuft cell morphology.

### Tuft Cell Core Actin Architecture

Tuft cell protrusions are supported by a cytoskeletal core comprised of many actin filaments that are bundled in parallel, creating an elongated linear structure that protrudes from the apical surface ~2 μm tall and 0.2 μm wide (Figure 1b). Ultrastructural studies suggest that tuft cells lack the meshwork of actin and intermediate filaments that provides a subapical anchoring point platform for enterocyte microvilli (33). Without this terminal web, tuft cell core actin bundles extend many microns deep into the cytoplasm, all the way to the perinuclear region, as long, cable-like structures (10, 33, 37) (Figure 1c).

Based on similarities in their morphology and cytoskeletal characteristics, tuft cell apical protrusions are most often compared to microvilli and stereocilia, which extend from the surface of solute transporting and mechanosensory epithelial cells, respectively (38,39). The large apical protrusions of tuft cells are easily identifiable against the background of shorter microvilli presented by neighboring enterocytes (7). Microvilli stand at ~1 μm tall and are ~0.1 μm in diameter and contain roughly 20 actin filaments bundled in parallel, which are tightly wrapped in apical plasma membrane. Microvilli rootlets extend into the terminal web, which is composed of cross-linked actin and intermediate filaments, just below the apical surface of the cell (40–43). Although the filaments that comprise a microvillus core do not run the full length of the bundle, electron microscopy (EM) cross-sections suggest that the number of filaments at the tip is comparable to the number observed in the rootlet (44). The large, tightly packed array of microvilli presented by enterocytes (~3,000 per cell) facilitates the nutrient absorption by increasing apical surface area at least tenfold (45). Stereocilia, on the other hand, are larger structures up to 5.5 μm long and 0.2 μm wide that are supported by actin cores consisting of hundreds of filaments in the distal part of the core, but fewer than 50 filaments at the base (39, 46). Although microvilli, stereocilia, and tuft cell protrusions are all supported by parallel bundles of F-actin, their structure and support of cellular functions are distinct.

On a subcellular level, actin bundles are assembled and stabilized through linking of individual filaments via actin-bundling proteins. Although these range in size and molecular structure, actin bundlers must be small enough to fit between actin filaments for dense packing and contain more than one actin-binding motif allowing them to bind multiple filaments simultaneously. Importantly, cell biological studies have revealed that the activities of multiple actin bundlers are responsible for the unique bundle architectures observed in distinct cell types. For example, the actin filaments in a microvillus are uniformly packed and contain actin bundlers such as fimbrin, villin, espin, and mitotic spindle positioning (47–50). In stereocilia actin filaments have liquid packing, allowing for variable spacing and an increased number of filaments bundling (51). This is made possible by the presence of multiple actin bundlers such as fimbrin, fascin, and espin, which have different lengths and geometrical constraints for bundling. In addition to determining filament organization, actin bundling proteins may help to elongate bundles by modulating the kinetics of monomer incorporation or disassembly (49). Actin-binding proteins might also impact filament stability and thus length by interfering with the binding of actin-severing proteins (52).

Although the specifics of how actin filaments in tuft giant core bundles are organized and bundled remain unclear, several studies have identified actin-binding proteins that may contribute to the architecture of these bundles.

Fimbrin (also called plastin), an actin cross-linking protein associated with stereocilia and microvilli, ruffles, and microspikes (48), is found along the length of the core actin bundles in tuft cells (32). Fimbrin is part of a large family of calponin homology domain–containing proteins, which contain other actin cross-linkers such as α-actinin (53). Proteins in this family have a conserved F-actin binding domain and an N-terminal headpiece that contains two sites homologous to the calcium-binding sites of calmodulin (54). Fimbrin-bound filaments were straight, polarized, and somewhat uniform in spacing with the distance between filament centers around 10–13 nm (47, 55). Remarkably, fimbrin was also localized along the basolateral margins in the tuft cell, which was unique to this cell type across multiple tissues (32). In tuft cells, fimbrin likely contributes to the straightness and rigidity of core actin bundles, although this idea remains untested.

Although fimbrins are expressed in many cell types (48), tuft cells also express other actin-binding factors that demonstrate more restricted, tuft cell–specific expression. One such factor is advillin, which exhibits high structural homology to villin (56), a microvillus actin bundler that is monomeric and exhibits calcium-dependent actin binding (47). In low concentrations of calcium, villin cross-links filaments, although the resulting structures are not as organized as bundles generated by fimbrin. However, in high concentrations of calcium, villin severs filaments, aiding in structural disassembly (57). Advillin shares the carboxyl terminal headpiece of villin required for actin bundling, as well as its six-domain structure, and maintains a 75% similarity to villin at the amino acid level (56). Advillin was first identified as enriched in tuft cells through RNA sequencing in the small intestine (58). Interestingly, earlier studies have also shown villin staining in tuft cell core actin bundles (11, 32), but it was later found that antibodies used to detect villin cross-react with advillin. When noncross-reactive antibodies were used, advillin was specifically present in tuft cells, whereas villin was only present in enterocytes (59). Based on this immunostaining, advillin is strongly enriched at the apical tuft, but also present along the full length of core bundles. The specificity of advillin to tuft cells in the gastrointestinal epithelium was further corroborated by in situ hybridization experiments, which found that advillin messenger RNA (mRNA) was specific to tuft cells, whereas villin mRNA was found along the entire intestinal surface epithelium (60). Advillin is also present in sensory neurons (61) and found along neurite shafts, filopodia, lamellipodia, axon terminals, and growth cones. In cells, advillin overexpression increased filopodia length (62), and in vitro experiments demonstrated that advillin possesses actin capping, severing, and nucleating properties similar to villin (59). In tuft cells, advillin likely contributes to the bundling and stabilization of core actin bundles, although its in vitro activities indicate that advillin might have more global effects on the actin cytoskeleton in tuft cells.

The F-actin and microtubule-binding protein girdin (63, 64) is also found in tuft cells. Girdin has an actin-binding site, a phosphatidylinositol phosphate-binding motif, and an Akt phosphorylation site near the carboxyl-terminal domain and forms oligomers through its amino terminal and coiled-coil region (65). While the unphosphorylated form is found in the enterocyte brush border, tuft cells are specifically enriched with girdin phosphorylated on Y1798, which localizes along core actin bundles (66). In cell culture studies, girdin is responsible for stress fiber formation and promotion of cell migration, and when phosphorylated by Akt (S1416), girdin localizes to the lamellipodia of migrating cells (63). Whether Akt or another tyrosine kinase is responsible for girdin phosphorylation in tuft cells and how phosphorylated girdin contributes to tuft cell cytoskeletal structure remain unknown.

Lastly, ankyrin, a protein that links plasma membrane to the underlying cytoskeleton (67), was found at the basolateral surface in both enterocytes and tuft cells (37). Ankryin binds many ion-translocating membrane proteins and voltage-dependent sodium channels in neurons and is thought to contribute to the immobilization of these channels at the cell surface (68). Ankyrin also contains binding sites for tubulin, spectrin, and membrane (69), and addition of the ankyrin microtubule-binding motif to tubulin resulted in the bundling of microtubules in vitro (70).

The unique complement of actin bundling and binding proteins found in tuft cells is probably critical for generating and maintaining tuft cell morphology. Based on the diversity of proteins that are now known to contribute to apical specializations such as microvilli and stereocilia, we expect that, beyond the few factors highlighted, more tuft cell–enriched cytoskeletal machinery will be uncovered in the near future. Elucidating the basic cell biological mechanisms underpinning tuft cell cytoskeletal organization and tuft morphology should be a priority for future studies.

### Microtubules and Intermediate Filaments

Tuft cells display a strong immunofluorescence signal for both alpha and beta tubulin in the apical cytoplasm compared to neighboring cells in the pancreatic duct and small intestine (37, 58). The microtubule network is oriented vertically in tuft cells (Figure 1a). Moreover, ultrastructural studies revealed that microtubules interdigitate between the core actin bundles, although they do not extend into the apical portion of the tuft where core bundles are wrapped with plasma membrane (10, 71). Tuft cell microtubules are also enriched in acetylated tubulin (11), a post-translational modification that contributes to microtubule stability by increasing flexibility (72). Additionally, doublecortin-like kinase 1 (DCLK1) is highly enriched in mouse tuft cells and has been shown to contribute to microtubule stabilization in other cell types (73). The enrichment of DCLK1 and acetylated tubulin likely promotes the stability of the microtubule network in tuft cells.

Tuft cells are also noted to be rich in intermediate filaments (10,74). Cytokeratin 18 is enriched in tuft cells (58), with intense labeling of the entire cytoplasmic portion of the cell body (37). Tuft cells also contain neurofilaments, which are ordered in parallel and, like microtubules, intermingle with the core bundles in the subapical cytoplasm (75). Neurofilaments are characteristic of mature neurons, and organization of intermediate filaments between parallel microtubules is likewise seen in neuronal axons (76). While the biological significance of intermediate filaments in tuft cells is currently unclear, we speculate that, based on other cell models, they probably provide further structural reinforcement and protect against cellular stresses (77).

### Cytospinules

Early EM studies of tuft cells noted the presence of a few (3–4) lateral microvilli, now referred to as cytospinules, which extend from the basolateral membrane up to 3 μm and make contact with neighboring cells (71). Cytospinules have a core of filaments and contain both villin and fimbrin, although the presence of these is considerably weaker than in the tuft (32). Recently, volumetric ultrastructure techniques have provided high-resolution three-dimensional images showing cytospinules piercing neighboring cells and making direct contact with their nuclei. Although vesicles were not observed in cytospinules, the distal tip is electron dense and often wrapped in the nuclear membrane of the cell in which it makes contact (7). The functions of tuft cell cytospinules are currently unknown. However, it is important to note that polarized epithelial cells do contain basolateral filopodia that make cell-to-cell contacts and aid in migration. Indeed, previous cell culture studies established that filopodia are marked by the tip localization of myosin 10 (78). Determining whether cytospinules contain myosin 10 and can be considered filopodia will be important to considering the distinction between cytospinules and filopodia and may provide insight into the functionality of these protrusions.

### Tuft Cell Plasma Membrane and Tubulovesicular Network

On the apical surface plasma membrane, tuft cells have a decreased protein-to-lipid ratio compared to enterocytes (33) and have a glycocalyx containing a different carbohydrate profile demonstrated by robust staining with *Ulex europaeus* agglutinin I (UEA-1) and *Lotus tetragonolobus* (LTA) lectins, which both detect l-fucose (79). Enterocytes, on the other hand, display a completely different lectin profile, with few lectins being statistically similar between both cell types. These lectins were also observed on electron lucent vesicles between apical tuft cell protrusions. Interestingly, a study of the adult rat tongue also exhibits strong UEA-1 staining (80).

Within the tuft cell, EM studies have identified numerous electron lucent vesicles in the apical cytoplasm, along the filamentous network previously described (7, 10, 33, 74). The dense array of vesicles and tubules in the tuft cell has indicated the presence of a tubulovesicular network that stretches from the apical surface of the cell down to the endoplasmic membrane (7). It seems likely that the tubulovesicular network functions to transport molecules, although interestingly, multiple studies have shown that tuft cells do not endocytose macromolecules (33). What molecules are being transported and whether the tubulovesicular network functions as part of the chemosensory role of tuft cells are currently unknown. Taken together these studies suggest that tuft cells possess a unique molecular framework to support their morphology.

## INTESTINAL TUFT CELL LINEAGE SPECIFICATION

Tuft cells are a morphologically distinct cell type; however, transcriptomic and functional heterogeneity exists, even within the same tissue. First suggested by Haber et al. in 2017 (81) and supported by several other groups, the most widely accepted tuft cell subtypes are tuft-1 and tuft-2 cells that are considered neuronal and immune, respectively (81–84). Neuronal, tuft-1 cells are broadly characterized by expression of choline acetyltransferase (ChAT), an enzyme required for synthesis of acetylcholine (ACh). Immune, tuft-2 cells express immune-related genes such as pan-immune marker CD45/*Ptprc*. Recently, SH2D6 and the G protein–coupled receptor (GPCR) VMN2R26 (vomeronasal type-2 receptor 26) were suggested as exclusive markers for tuft-2 cells, while TPPP3 is exclusive for tuft-1 tuft cells (85). Interestingly, tuft-1 and tuft-2 cells both express IL-25 (81). The specific functions of tuft-1 and tuft-2 cells have yet to be elucidated, but it is thought that tuft-2 cells are involved in immune responses to commensals and pathogens (85), while tuft-1 cells provide cholinergic signaling to support intestinal stem cells (84, 86). Although markers and functional effectors of these subpopulations have been identified, the specification and maintenance of each subpopulation remain unclear. Still, not all studies of tuft cell heterogeneity support the tuft-1 and tuft-2 cell subtypes (83, 84, 87). Subpopulations of tuft cells also have distinct characteristics outside of their transcriptional identities. For instance, neuronal tuft cells were found more often at the lower portion of the crypt around less-differentiated cells, compared to immune-like tuft cells that are found at the luminal surface (88).

Tuft cells also have distinctive lifetimes, with many turning over in ~7 days (89), whereas others are extremely long-lived, with lifetimes measured in years (90). This leads to questions regarding the developmental origins of different populations of tuft cells. Tuft cells were originally classified as a secretory cell type under the control of the master secretory transcription factor ATOH1 (15), but later studies found that tuft cells can still be specified without ATOH1 (91, 92). It was further revealed that ATOH1-independent specification of tuft cells requires extrinsic stimuli such as the presence of microbiome, while ATOH1 is intrinsically required without stimuli (18). Interestingly, colonic tuft cells are ATOH1-dependent while the small intestine contains a mix of dependencies. SOX4 has also been identified as a key transcription factor in tuft cell specification regardless of ATOH1 activity (93). Moreover, seminal work has now identified POU class 2 homeobox 3 (POU2F3; *Skn-1a*) as a master transcription factor that induces features of all chemosensory cells, including lingual taste cells and tuft cells (20, 94).

**Figure 2:**
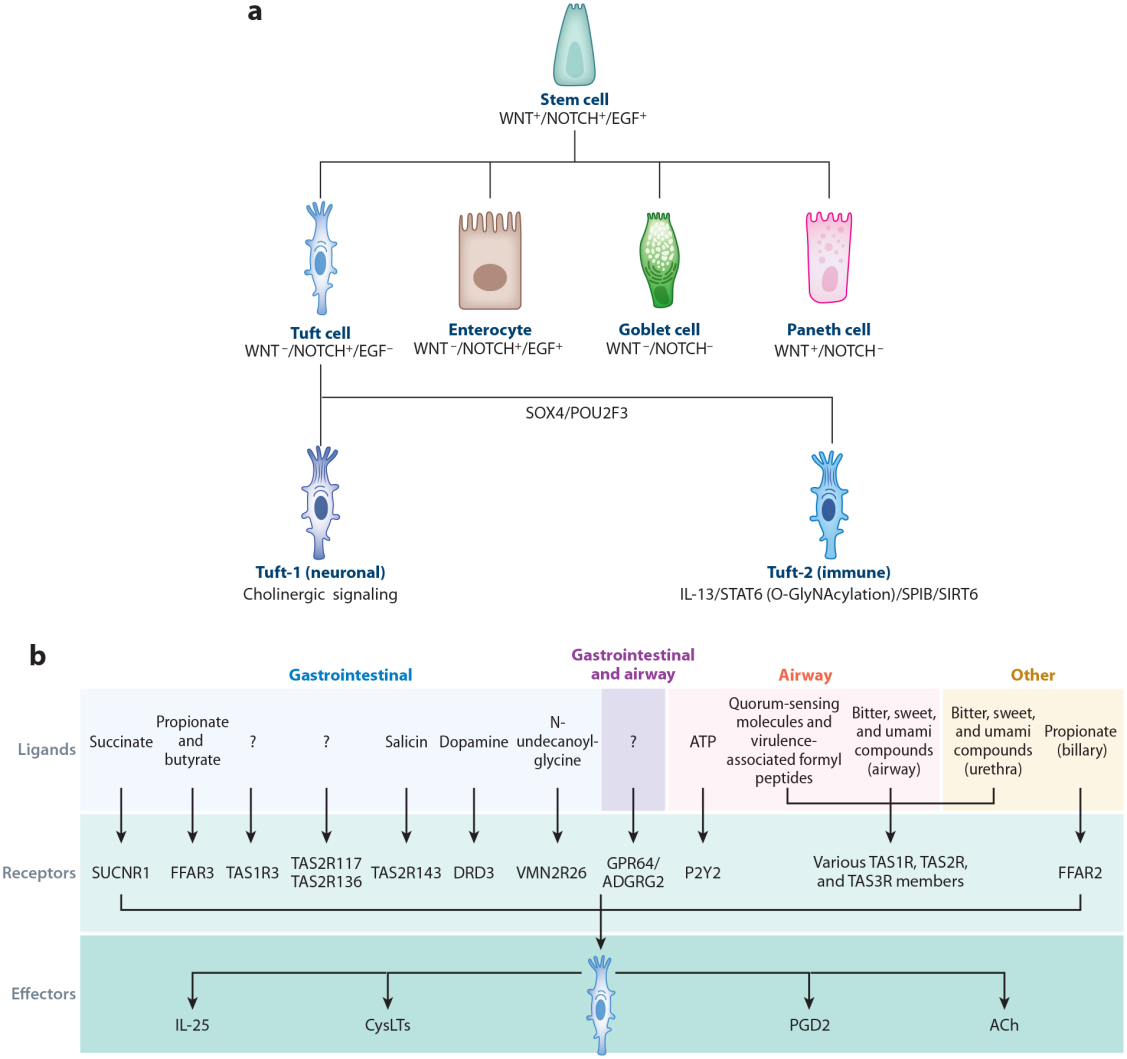
(*a*) Illustration depicting signal code for epithelial cell differentiation and specification of tuft-1 and tuft-2 cells. (*b*) Graphic depicting the current knowledge of organ-/tissue-specific tuft cell ligands and their corresponding receptors. Tuft cell effectors that are downstream of many of these ligand-receptor interactions are listed below. Abbreviations: ACh, acetylcholine; CysLT, cysteinyl leukotriene; DRD3, dopamine receptor D3; EGF, epidermal growth factor; FFAR2, free fatty acid receptor 2; FFAR3, free fatty acid receptor 3; GPR64/ADGRG2, adhesion G protein–coupled receptor G2; IL-25, interleukin 25; PGD2, prostaglandin D_2_; P2Y2, P2Y purinoceptor 2; POU2F3, POU class 2 homeobox 3; SIRT6, sirtuin 6; SOX4, SRY (sex determining region Y) box 4; SPIB, Spi-B (transcription factor); STAT6, signal transducer and activator of transcription 6; SUCNR1, succinate receptor 1; TAS1R, taste 1 receptor; TAS1R3, taste 1 receptor member 3; TAS2R, taste 2 receptor; TAS2R117, taste 2 receptor member 117; TAS2R136, taste 2 receptor member 136; TAS2R143, taste 2 receptor member 143; TAS3R, taste 3 receptor; VMN2R26, vomeronasal type-2 receptor 26; WNT, wingless/integrated, NOTCH, neurogenic locus notch homolog protein.

Additionally, the signaling code for epithelial cell differentiation from stem cells has been well established: WNT and NOTCH activation maintains stemness, NOTCH alone promotes absorptive differentiation, WNT alone promotes Paneth cell differentiation, and absence of NOTCH and WNT signaling promotes secretory cell differentiation (Figure 2a). Organoid studies by Hans Clevers’ group (95) revealed the contribution of EGF signaling to this code. In the study, absorptive differentiation is promoted by NOTCH and epidermal growth factor (EGF) signaling in the absence of WNT, while tuft cell differentiation is promoted by NOTCH alone without WNT and EGF. The involvement of NOTCH signaling is also seen in the differentiation of tuft cells from ectopic basal cells in the lungs. Tuft cell differentiation is promoted by WNT inhibition, whereas it is suppressed by NOTCH inhibition (96). Consistent with the requirement of EGF signaling inhibition on differentiation, increased epidermal growth factor receptor (EGFR) endocytosis and activation of downstream mitogen-activated protein kinase (MAPK) by overexpression of cell division control 42 (CDC42) isoform V2 reduced intestinal tuft cell specification by 80% (97). Further studies revealed that DDX5 promotes the translation of CDC42 and expression of protein and lipid metabolism gene programs in tuft cells. DDX5 deletion in intestinal epithelial cells impairs tuft cell specification downstream of SRY (sex determining region Y) box 4 (SOX4+) progenitors independent of IL-13 and succinate stimuli (83).

Type-2 immune signaling in response to eukaryotic parasites through IL-13 and the downstream transcription factor signal transducer and activator of transcription 6 (STAT6) is another significant signaling pathway leading to tuft cell specification (21, 98). Newer work has demonstrated that STAT6 can be modulated to impact tuft cell differentiation. For instance, epithelial O-GlyNAcylation of STAT6 is required for its activity in driving POU2F3-dependent transcription and differentiation of tuft cells in response to parasites and expression of GSDMC for further IL-33 secretion (99). Sirtuin 6 (SIRT6) deletion induces SOCS3 expression, negatively regulating tyrosine phosphorylation of epithelial STAT6 and tuft cell differentiation in response to helminth infection (100). SIRT6 deletion also reduced the chromatin accessibility of POU2F3-regulated genes, decreasing the number of thymic tuft cells (101). Organoid studies revealed that tuft cells express BMP ligands inducible by IL-13, which forms a negative feedback loop to limit tuft cell differentiation by downregulating SOX4 in stem and progenitor cells (102). In addition, immune-related CD45+ tuft cells that fight bacterial infection have been shown to be specified in response to bacterial metabolite-induced VMN2R2 signaling to the Spi-B (SPIB) transcription factor (85).

In contrast, work on epithelial-enteric nervous system interactions has shed light on compensatory neuronal tuft cell specification. The requirement of cholinergic signaling for tuft cell specification or maintenance was demonstrated in organoids, where tuft cells are lost unless cocultured with neurons or supplemented with cholinergic agonist (90). Interestingly, ablation of muscarinic receptor, part of the cholinergic signaling pathway, induces tuft cell specification as a function of decreased EGF signaling and activated phosphoinositide 3-kinase (PI3K)-phosphoinositide-dependent kinase-1 (PDK1) signaling in epithelial cells (103). These tuft cells are of the neuronal subtype, share a common PROX1^+^ progenitor with enteroendocrine cells (104), and express ChAT to produce acetylcholine, compensating for the cholinergic signaling deficits that are required for their specification or maintenance (103). Consistent with the above findings, epithelial inositol polyphosphate multikinase (IPMK), critical in the production of inositol polyphosphates, has also been shown to be involved in cholinergic tuft cell specification. Neuronal tuft cells reside in the crypt and interact with stem cells that facilitate recovery from colonic injury (84). SPROUTY2 loss also increases PI3K/Akt signaling, leading to intestinal tuft and goblet cell expansion, and SPROUTY2 downregulation appears to be protective in the context of epithelial damage and inflammation (105).

Aside from EGF, immune, and neuronal pathways, recent work has also shed light on other genes that regulate tuft cell abundance and specification. Several groups have identified cofactors that boost POU2F3 transcription factor activity to induce tuft cell specification. Two homologous proteins with previously unknown functions, now named OCA-T1/POU2AF2 and OCA-T2/POU2AF3, were found to have similar expression patterns as POU2F3 (106). These proteins serve as transcriptional activators and bind POU2F3 to maintain chromatic accessibility and enhancer activity to drive tuft cell specific gene expression (107). Alternative isoforms of POU2AF2 were recently identified via genetic mapping of different mice strains, with its short isoform encoding a nonfunctional protein that leads to decreased tuft cell specification (108). Finally, in separate studies, tuft cell abundance is reduced upon deletion of TAS1R3, a taste GPCR, or Raptor/RPTOR, a component of the mechanistic target of rapamycin complex 1 (mTORC1) pathway (109, 110). It remains unclear whether these genes alter the specification or maintenance, or other components, of tuft cells.

## INTESTINAL TUFT CELL RECEPTORS AND CORRESPONDING LIGANDS

The unique, elongated morphology of the apical protrusions on intestinal tuft cells poises them for superior chemosensory function and contact with luminal ligands. Across organs and tissues, tuft cells express context-specific taste and olfactory receptors and other GPCRs, a subset of which are expressed by intestinal tuft cells (58, 81, 111–113) (Figure 2b). The most widely studied intestinal tuft cell receptor is succinate receptor 1 (SUCNR1), a GPCR that is engaged by microbial or helminth-derived succinate ligand (17, 112, 114). The GPCR GPR64/ADGRG2 is expressed in tuft cells in airway and gastrointestinal organs, including the intestine, and genetic ablation of GPR64 resulted in complete tuft cell depletion in the intestine (113). Free fatty acid receptor 3 (FFAR3), a GPCR that detects the microbiota-derived short-chain fatty acids propionate and butyrate, is also expressed in small intestinal and colonic tuft cells (84, 112, 113). Another GPCR and dopamine receptor, dopamine receptor D3 (DRD3), has been detected in intestinal tuft cells (81, 113). Among TAS1R taste receptors, which were originally characterized as lingual sweet and umami receptors, only TAS1R3 is expressed in intestinal tuft cells and at a higher abundance in the ileum (110). Ligands for TAS1R taste receptors on intestinal tuft cells have been difficult to identify, as microbial sources of ligands and dietary sugar substitutes (i.e., sucralose and steviol glycosides) have been ruled out. Gene expression profiling of 35 murine TAS2Rs revealed that *Tas2r117*, *Tas2r136*, and *Tas2r143* are expressed in intestinal tuft cells, and moreover, the bitter compound salicin serves as a ligand for TAS2R143 (115). Subtypes of intestinal tuft cells also express the olfactory receptor VMN2R26 and detect the ligand N-undecanoylglycine, a metabolite derived from *Shigella* (85, 113). The contribution of individual signal transduction components (i.e., TRPM5, PLCB2, ITPR3, and G protein subunits) that are involved downstream of ligand-receptor engagement has been investigated, and their requirement is context-dependent, based on the specific ligand, helminth, or microbe (17, 21, 110, 112, 115). However, the transcriptional signature from tuft cells across multiple tissues suggests GPCR-induced intracellular signal transduction is a common function of this specialized cell type, including responses to B cells, antibodies, and phosphatidylinositol (112). The calcium flux that occurs downstream of GPCR signaling likely triggers the expression and release of multiple tuft cell–derived effectors such as IL-25, acetylcholine, leukotrienes, and prostaglandins (22, 86, 116–119). While ATP is another effector that is released in response to canonical GPCR signaling, release from tuft cells has not been observed.

Although the understanding of ligand-receptor signaling in intestinal tuft cells is limited, other potential ligands and receptors can be surmised from knowledge in other tissues. Bitter, sweet, and umami compounds serve as taste receptor ligands that activate calcium signaling in tuft cells in the airway and urethra (120–122). Intriguingly, in the airway, this process can be abrogated by coadministration of sweet compound ligands glucose, sucrose, or sucralose, or by bacteria-derived d-amino acids that bind TAS1R receptors. Quorum-sensing molecules and virulence-associated formyl peptides from bacteria are detected by airway tuft cells by an unknown receptor (117,123–125). Airway tuft cells also detect ATP via the P2Y purinoceptor 2 (P2Y2) receptor (118). The bacterial fermentation product propionate acts as a ligand for the free fatty acid receptor FFAR2 expressed on biliary tuft cells, which, unlike intestinal tuft cells, are nonresponsive to succinate (126). Whether these ligands are relevant to intestinal tuft cells remains to be explored.

The exaggerated apical protrusions on tuft cells led investigators to focus on luminal ligands and apical receptors, but the possibility of basal receptors and stroma-derived ligands remains unexplored.

## INTESTINAL TUFT CELL OUTPUTS AND EFFECTOR FUNCTIONS

The most extensively characterized tuft cell effector molecule is IL-25, a cytokine that activates ILC2s in the type 2 immune response, initiating a feed-forward loop to increase tuft cell abundance (20–22). Tuft cell–derived IL-25 has been extensively covered in other reviews (19, 23–31). Here, we focus on more recent work that has uncovered several other intestinal tuft cell products and, in general, implicates tuft cells in cholinergic signaling and inflammation.

Tuft cells produce several synthases that are necessary for prostaglandin production, including cPLA2/*Pla2g4a*, COX1/*Ptgs1*, COX2/*Ptgs2*, and HPGDS/*Hpgds* (12, 15, 58, 81, 85, 127). Intestinal tuft cells were identified as a critical source of prostaglandin D2 (PGD2), as knockout of tuft cells depleted PGD2, while induced tuft cell hyperplasia increased PGD2 levels (128). Moreover, this process is inducible, as tuft cells produce PGD2 in response to the intestinal pathogen *Shigella* (85). Intestinal tuft cell–derived PGD2 acts on intestinal epithelial cells to counteract antiproliferative signals induced by type 2 cytokines (128).

Cysteinyl leukotrienes (CysLTs) are inflammatory lipid signaling molecules that were first identified as products of hematopoietic cells that potentiate inflammation (129). However, several groups reported expression of enzymes that synthesize CysLTs (i.e., ALOX5, LTC4S, PLA2G4A) in intestinal and nonintestinal tuft cells (58, 81, 87, 112, 118, 126). Tuft cell–derived CysLTs potentiatetheIL-25-mediated activation of ILC2s that induces tuft cell hyperplasia during antihelminth responses in the small intestine (116). Interestingly, CysLTs were not required for antiprotist responses. While our knowledge of intestinal CysLTs is still being uncovered, findings in other organs suggest CysLTs may be involved in more than just inflammatory processes. For example, biliary tuft cell CysLTs mediate gallbladder constriction, whereas airway tuft cells generate CysLTs in response to ATP from aeroallergens (126).

Another ubiquitous tuft cell marker across tissues is ChAT, an enzyme that synthesizes the cholinergic neurotransmitter ACh (103, 112, 130). In the intestinal epithelium, tuft cells are the exclusive source of ACh, which is an important regulator of WNT signaling that supports the intestinal stem cell niche (86, 131, 132). ACh also mediates intestinal contraction in weep and sweep responses that are important for helminth clearance (133). Recently, tuft cell–derived ACh was demonstrated to elicit fluid secretion from epithelial cells in the intestine and airway in response to tuft cell chemosensation of succinate or by direct modulation of taste receptor signaling (132). In biliary tuft cells, ACh does not mediate gallbladder constriction, but instead, promotes goblet cell mucous secretion (126). In contrast, ACh produced by tuft cells in the airway or urethra signals to neurons to trigger organ-specific reflexes (120, 123, 124). This cholinergic signaling process is also linked to neurogenic inflammation (134). Like CysLTs, the full picture of the role of intestinal tuft cell–derived ACh remains unknown. Curiously, expression of choline uptake transporters (CHT1/*Slc5a7*) thought to be required for ACh synthesis, as well as vesicular ACh transporters (VAChT/*Slc18a3*), has not been observed in tuft cells (130).

## INTESTINAL TUFT CELL CONTRIBUTIONS TO BARRIER MAINTENANCE

Tuft cells engage in the weep and sweep to remove eukaryotic parasites through cooperation with other intestinal epithelial cell types. To date, our knowledge of tuft-enterocyte cooperation is limited to a recent study demonstrating that tuft cell effector ACh stimulates enterocyte fluid secretion (132). In contrast, coordination of tuft cells with other intestinal epithelial secretory cells is more established, such as enhanced differentiation of mucus-producing goblet cells (20–22). Because secretory cells play major roles in maintaining the physical and chemical barrier of the gut, tuft cells can modulate the microbiome indirectly by augmenting the antimicrobial capacity and function of these cell types, and vice versa.

Succinate, derived from the microbiota or administered exogenously, induces increases in goblet cell size and proportion, and this process is dependent on tuft cell expression of the succinate receptor SUCNR1, but independent of expression of taste transduction signaling components α-gustducin (GNAT3) or TRPM5 (17). This process mirrors what was previously established in the antihelminth-induced expansion of goblet cell proportion and size via tuft cell–dependent responses (20–22, 135, 136). Intestinal colonization with the gut commensal protist *Tritrichomonas musculis*, or succinate administration, activates tuft cells and induces type 2 immunity that alters the antimicrobial function of the intestinal epithelium (137). These alterations include a decrease in some antimicrobials, such as lysozyme- and Paneth cell–derived defensins, but an increase in others, such as resistin-like beta (RETNLB). Tuft cell–originated alterations in antimicrobial expression from secretory cells resulted in changes to the composition and overall reduction of mucosa-associated bacteria, although changes in the luminal bacterial load were not detected. Of note, these antimicrobial changes can be induced by bypassing tuft cells and ILC2s through direct administration of cytokines IL-25 or IL-13. Another example of tuft cell–mediated microbial response is the detection of *Shigella* by tuft cell–specific receptors, resulting in the expansion of tuft cells and PGD2 production, leading to an increase in goblet cell mucus production to control infection (85). Although these studies suggest that tuft cells initiate changes to the antimicrobial landscape by altering secretory cell function, tuft cells are also responsive to alterations to secretory cell function and the consequential microbial changes. For example, loss of Paneth cell–derived lysozyme results in alterations of the microbial landscape and increased tuft cell proportions (138). Similarly, loss of the intestinal epithelial secretory cell lineage results in an increased abundance of ATOH1-independent tuft cells in the small intestine (18).

## TUFT CELLS IN INTESTINAL DISEASES

### Intestinal Inflammation

Given the role of tuft cells as a conduit between the luminal environment and host, as well as their roles in shaping the microbiome landscape, it is no surprise that tuft cells also impact several disease processes in the gut. Human data in addition to experimental perturbations in mouse models support an anti-inflammatory role for tuft cells in intestinal inflammation. In human inflammatory intestinal diseases, such as celiac disease and duodenitis in pediatric patients (139), ileal Crohn’s disease (18), and quiescent ulcerative colitis (140), tuft cell abundance is diminished when compared to healthy controls. Moreover, ileal inflammatory disease in mouse models can be attenuated in a tuft cell-dependent manner, for instance, by inducing their expansion by administering the bacterial metabolite succinate (18, 83). While the effects of tuft cell barrier regulation and the immune response play significant roles in countering inflammation, their role in boosting the regenerative capacity of intestinal stem cells is also important in restitution from epithelial damage. In the colon, tuft cell expansion promotes, while their depletion diminishes, stem cell–driven restitution after epithelial damage induced by radiation or dextran sodium sulfate (DSS) (83, 84, 86, 90, 105, 141, 142). The anti-inflammatory role of tuft cells can also be observed in the pancreas, where tuft cells rising from pyloric metaplasia (12, 143) restrict pancreatic tumorigenesis by suppressing immune activation and fibrosis (127).

### Intestinal Cancer

The anti-inflammatory role of tuft cells may contribute to their pro-tumorigenic role in the gut. Tuft cells and their markers are found within several human tumors and dysplastic lesions, including colorectal tumors, and are correlated with poor prognosis (11, 15, 89, 90, 144–153). In mice, tuft cell depletion in the *Apc*^*flox/*+^ intestinal tumorigenesis model results in a decreased tumor burden, while succinate administration restores DCLK1^+^ tuft cells and increases tumor burden (83, 154). In the *Apc*^*Min/*+^ model, downregulation of DCLK1 expression, a tuft cell marker, also led to reduced polyp formation and dysplasia (148). In this context, DCLK1^+^ cells enhance pro-survival signaling to promote tumorigenesis (148). While there is debate whether tuft cells act as intestinal stem cells at homeostasis (90), it is accepted that tuft-like cells can act as tumor stem cells. In the *Apc*^*Min/*+^ model of intestinal tumorigenesis, DCLK1^+^ and IL-17RB^+^ tuft-like cells lineage trace within tumors (89, 151). When these tumor cells are ablated, tumor growth is suppressed in vivo. *Apc* deletion specifically in DCLK1^+^ cells can initiate tumors when combined with DSS damage (90), further supporting the role of these cells as tumor initiating cells in the gut. In small cell lung cancer, tuft-like cells constitute an entire subtype of tumors, and this phenotype can be propagated in cell culture, demonstrating these cells’ renewal capacity (155). In addition to tuft cells’ inherent stem properties in tumors, tuft cell-derived IL-25 also promotes stemness in colorectal tumors, suppresses antitumor immunity, and is correlated with poor prognosis in patients (156, 157).

## VIRAL INFECTION IN THE INTESTINE AND TUFT CELL TROPISM

Tuft cells may be a strategic viral host cell owing to their exaggerated apical protrusions and potential for being a long-lived reservoir (90). Viral tropism for intestinal tuft cells was first demonstrated with murine norovirus (MNV), where tuft cells are the exclusive epithelial target (122, 158). MNV entry is mediated by the CD300LF receptor expressed by tuft cells, while persistence is mediated by interferon signaling from nonepithelial cells (31, 158).Moreover, modulation of the tuft cell population is correlated with MNV infection (158, 159). Although immune responses appear intact, infected tuft cells are uniquely insulated from immune clearance, which allows MNV to persist (159–161). Of note, CD300LF is not a receptor for human norovirus, and human tuft cell tropism has not been demonstrated (162). Thus far, MNV is the only virus demonstrated to have exclusive tropism to intestinal tuft cells; however, MNV was recently demonstrated to infect intestinal tuft cells along with the villus-tip enterocytes (163). Transcriptomics of rotavirusinfected intestinal tuft cells suggest that tuft cells respond by downregulating canonical effector functions, such as IL-25 secretion, and instead upregulate chemosensory genes (163). Intestinal tuft cells also play an indirect role in intestinal viral infection in the context of coinfection with helminths. Helminths increase tuft cell proportions even in the context of viral coinfection, but intriguingly, this is associated with a dampened CD8^+^ T cell response (158, 164, 165). While not tuft cell tropic, flaviviruses increase their viral burden in the context of helminth coinfection by indirectly taking advantage of tuft cells, resulting in increased intestinal permeability and destruction of virus-specific CD8^+^ T cells (165).

Studies of viral infections in non-intestinal tissues may elucidate additional tuft cell tropism within the intestine. Patients with COVID-19 have emergent populations of tuft and tuft-like cells in the airway (166). Additionally, in many airway bulk transcriptomic data sets, expression of the SARS-CoV-2 receptor, ACE2, and the tuft cell marker, POU2F3, are positively correlated (166–177). Single-cell data from the respiratory tract reveal that tuft cells express higher levels of ACE2 receptor compared to other respiratory epithelial cells, supporting the possibility of SARS-CoV-2 tropism for airway tuft cells (177). This is a particularly intriguing finding, given the chemosensory function of tuft cells in the airway and the frequent loss of taste and smell in COVID-19 patients. While intestinal absorptive enterocytes express ACE2, intestinal tuft cells do not express the receptor, suggesting that if SARS-CoV-2 has tuft cell tropism, it is likely restricted to airway tuft cells (97). Murine airway infection models have also been used to elucidate the role of tuft cells in viral infections; however, additional viral tropism for tuft cells has yet to be reported. As observed in human respiratory infection, heterogeneous populations of tuft cells are also generated de novo in the murine airway after severe viral infection, such as influenza or COVID-19 virus (96, 178, 179). Intriguingly, these induced tuft cells arise independent of type 2 cytokines. Moreover, tuft cell–depleted mice have less inflammatory infiltrate in the lungs during influenza infection compared to wild-type mice, suggesting that tuft cells mediate an inflammatory response to viral infection (166). Across mouse and human, the presence of tuft cells in the lung does not seem to impact regeneration, differentiation, and repair after injury. More work needs to be done to understand the role of tuft cells in other models of mucosal injury and inflammation. Interestingly, tuft cell abundance in the intestine increases during respiratory influenza infection; however, their presence or absence had no effect on disease course (180).

## OTHER DISEASE PROCESSES

Intestinal tuft cells have been implicated in other intestinal disease processes and extraintestinal diseases. Tuft cells are also lost in models of microvillus inclusion diseases, a condition caused by maldifferentiation of enterocytes through loss of MYO5B or CDC42 (97, 181). Tuft cells can be rescued in these models by correcting the differentiation defect, for example, by rebalancing epithelial WNT and NOTCH signaling.

Intestinal tuft cells are also implicated in various metabolic diseases. The relative proportion of tuft cells in the small intestine was increased in mice that were fasted and remained elevated after refeeding (16). Studies in mice on a high-fat diet and with high-fat diet-induced obesity also implicate tuft cells in metabolic processes (182). MNV infection protects mice from the development of type 1 diabetes, and given the tropism for tuft cells, future studies on the role of tuft cells in this metabolic disease are warranted (183).

Finally, intestinal tuft cells play roles in skin allergic responses and chronic inflammation. Mechanical skin injury induces changes in the intestine, including an increase in tuft cells and IL-25 and subsequent activation and expansion of intestinal ILC2s (184). Similarly, mechanical skin injury with peanut antigen challenge resulted in elevated succinate levels in circulation, increased tuft cell abundance and IL-25 levels in the intestine, and a heightened type 2 response (185).

## DISCUSSION

### Tuft Cell Therapeutics in Human Health

Given the connection of DCLK1 expression and tuft cell abundance in human tumors, several tuft cell–targeted inhibitors were developed and tested in various models. DCLK1 inhibition is an appealing target, as it is expressed in malignant cells but absent in normal human tuft cells. MicroRNAs and small-molecule inhibitors of DCLK1, LRRK2-IN-1, and DCLK1-IN-1 show promise against colorectal and pancreatic cancer cells in vitro, and DCLK1-targeting CAR-T cell (chimeric antigen receptor T cell) immunotherapy reduces tumor growth in murine models of colorectal cancer (147, 186–192). While DCLK1 inhibition is effective in translational models, inhibitors have not been tested in clinical trials due to concerns regarding the druggability of DCLK1 and its lack of specificity to tuft cells remain. Other tuft cell markers are also unattractive candidates for therapeutics due to expression in additional tissues and cell types. For example, taste receptors are expressed in chemosensory cells in multiple organs and are known to regulate critical metabolic processes (193). TRPM5, a downstream component of taste receptor signaling, is also expressed in pancreatic beta cells and is critical to glucose-induced insulin release (194). Neutralization of ligands that are chemosensed by tuft cells could be an interesting approach, but few tuft cell ligands have been revealed in the intestine. Succinate is the most extensively characterized; however, this metabolite also serves as a key component of the tricarboxylic acid cycle and is involved in inflammatory processes. Thus, neutralization of succinate would likely have unwanted systemic effects. In contrast, IL-17RB, a component of the IL-25 receptor, has emerged as a promising candidate because it is expressed on tuft cells and colorectal tumor stem cells (151). In support of its druggability, several other interleukin receptor antagonists have been developed and are currently used to treat a variety of human diseases.

### Tuft Cells from Mouse to Human

Murine studies have elucidated many markers and molecular features of tuft cells that tell us more about their structure and function, but we know that not all features are shared in human tuft cells. A concise table of tuft cell markers that are shared across mouse and human has been prepared in other reviews, most recently by Strine & Wilen in 2022 (31). In general, human and murine tuft cells express common sets of transcription factors, enzymes required for prostaglandin, acetylcholine, and leukotriene synthesis, factors for intracellular taste receptor signaling transduction, and cell surface receptors. Notable differences include the lack of expression of DCLK1, SIGLECF, and IL-25 in human tuft cells. Given the importance of IL-25 on type 2 immune responses, the lack of human tuft cell IL-25 expression should be further investigated and supports the notion that tuft cell adaptations account for their acquired immune function. Moreover, while IL-13 induces a robust expansion of intestinal tuft cells by signaling to stem cells, this has yet to be replicated in human organoids. Studies in human colorectal cancer organoid lines revealed that while tuft cells were present in a small proportion of established lines, IL-13 was dispensable for their maintenance (151). Within the human small intestine, the ultrastructure of tuft cells was comparable to those found in other animal models (36). Although certain components such as cytokeratin 18 and advillin were found in analogous locations within human and murine tuft cells (195), a comparative study looking at all tuft cell morphological and cytoskeletal features has yet to be completed.

### Connection of Architecture to Function

Although tuft cells have been studied for decades, research on basic tuft cell biology has lagged behind the elucidation of tuft cell physiological function. This is largely due to technical limitations including tuft cell rarity in native tissues and the lack of representation of tuft cells in epithelial cell culture models. Despite the intimate relationship between cell morphology and function, how the unique morphology of the tuft cell contributes to its role in the epithelium of the gastrointestinal tract and hollow organs remains unclear. Surprisingly, tuft cells from different tissues are strikingly similar in morphology despite significant heterogeneity in gene expression profiles and function. Indeed, the signatures between tuft-1 and tuft-2 cells are strong, yet no significant structural differences between these groups have been noted; some evidence suggests that succinate stimulation does not impact vesicles or the cytoskeletal ultrastructure of intestinal tuft cells (196). These results and others point to a global application for tuft cell architecture that transcends tuft cell function and location.

While giant apical protrusions and their supporting core actin bundles are defining features of tuft cell morphology, we have little information on how these structures contribute to tuft cell function. However, based on studies in other systems, we can offer some informed speculation, and here we focus on three possibilities: extracellular vesicle release, polarized trafficking, and mechanosensation.

Tuft cell apical protrusions are twice as long as microvilli, which allows them to reach farther into the luminal space, potentially breaching the layer of mucus that covers the epithelium. Given that the mucus layer is intended to exclude parasites, the elongated nature of tuft cell protrusions might allow these cells to sample or respond to a wider range of stimuli and may allow tuft cell–derived vesicles and secreted molecules access to the lumen. Vesicles between tuft cell apical protrusions were found to have tuft cell–enriched lectin staining (79), suggestive of extracellular vesicles. Many cells generate extracellular vesicles (197) and an interesting example is provided by enterocytes, which use microvilli to release extracellular vesicles enriched in intestinal alkaline phosphatase into the lumen (198). Electron-dense, nonmembrane-wrapped granules have also been observed between tuft cell apical protrusions near the apical surface (199), pointing to an alternate form of secretion for these granules. Electron-lucent vesicles were also identified at the basal surface of tuft cells (199), although the identity of these vesicles remains unknown. While ACh is a common tuft cell effector, tuft cells lack VAChT, which packs ACh into vesicles, suggesting an alternate mechanism of ACh secretion in tuft cells (130). On the other hand, IL-25 secretion is reduced when villi are pretreated with brefeldin A, which inhibits endoplasmic reticulum to Golgi transport, suggesting that IL-25 is released through vesicles (115). The identification of the vesicular cargo is critical to inform our understanding of how the cytoskeletal network in tuft cells functions. Common tuft cells are good targets for determining vesicle identity, but there may be additional targets for cargo released into the lumen.

Tuft cell actin core bundles extend from these apical protrusions down into the perinuclear region and interdigitate with a network of microtubules in the intervening cytoplasm. Actin filaments are polarized polymers with structurally distinct barbed and pointed ends; incorporation of new actin monomers is favored at the barbed ends, the process of which has been extensively reviewed (200). In polarized cells such as microvilli, the plus end of actin and the minus end of microtubules oriented toward the apical membrane (201, 202). Although this point has not been clarified experimentally, studies in microvilli and stereocilia lead us to speculate that tuft cell core bundles probably contain filaments that are aligned in parallel, such that all barbed ends extend toward the plasma membrane. If this is the case, these structures would be able to support directed trafficking between the perinuclear and apical compartments, presumably driven by myosin motor proteins. Studies in other systems provide many examples of cargo trafficking along polarized bundles of actin filaments. Indeed, myosin 10 has been shown to deliver actin elongation factors such as vasodilator-stimulated phosphoprotein to the tips of filopodia (203); in stereocilia, whirlin and EPS8 may be delivered to the tips of stereocilia by myosin-15 (204), whereas espin-1 has been identified as a cargo that is trafficked to the tips by myosin-3a and myosin-3b (205). Microtubules are involved in many apical delivery processes, a specific example of which is seen in the apical trafficking of neurotrophin receptor p75 by microtubule motor kinesin KIF5C (206). In tuft cells, we speculate that the interdigitating combination of microtubules and polarized actin bundles may serve as a “superhighway” for vesicle transport to and from the apical domain.

Another possibility is that the core actin bundles in tuft cells act as mechanosensors. As a point of comparison, stereocilia are considered a primary example of mechanosensory actin structures. Critically, a single stereocilia is not mechanosensitive on its own. Rather, a deflection of one stereocilia creates a force on the linkages between protrusions, opening critical ion channels in the cell (207). Stereocilia have very small rootlets that function to fasten the bundle in the cuticular plate (46), but in tuft cells, it is possible that the long rootlets serve to propagate the mechanosensation deep into the cell and stimulate a response. The tall apical tuft is likely subject to luminal pressures as well as direct forces from parasites. Helminths are large parasites, up to 1 mm in length, and the mechanical force generated by contact with the parasite would cause a deflection in the large apical protrusions and core actin bundles that may in turn contribute to the tuft cell response. Although tuft cell immune response can be stimulated in the absence of parasites using the metabolic byproduct succinate (18), it is unknown whether mechanical perturbation may be an additional trigger.

### Tuft Cell Models

Tuft cells remain a difficult cell type to study due to both their rarity in tissue and a lack of representation in cell culture models. Knockout mouse models may be a particularly useful tool for understanding the impact of a specific molecule on tuft cell structure. However, the compensatory ability of the epithelial tissues can obscure the function of specific molecules. For example, enterocytes in a triple knockout of three microvilli proteins—villin, espin, and fimbrin—still produced microvilli, although the filament density and organized packing in the actin bundles were lost (208). Cell culture and in vitro data also provide valuable information. For instance, advillin knockout in adult dorsal root ganglia found a decrease in neurite length and branching and impaired neurite regeneration (62). However, how a loss of advillin impacts tuft cells is currently unknown. Some functionality may be revealed only under stimulation or stress. In the case of girdin-depleted human vesicular smooth muscle cells, no phenotype was observed until the cells were stimulated, at which point stress fibers became disrupted and lamellipodia size decreased (65). Generation of Cre drivers from tuft cell–specific markers enables precise genetic targeting of tuft cells, facilitating visualization and permitting genetic manipulations that can be used to investigate their function. For instance, constitutive and/or inducible Cre drivers have been generated from *Dclk1*, *Pou2f3*, *Sh2d6*, *Avil*, and *Chat* promoters and have proven to be powerful tools for studying tuft cell biology (24, 60, 85, 89, 90, 116, 209, 210).

Organoids generated from mammalian tissues provide another avenue for studying tuft cells, as they can recapitulate multilineage differentiation in an experimentally tractable setting. We are just scratching the surface of knowledge that can be gleaned from these platforms, as previous studies have only examined basic tuft cell outputs by staining tuft cell–specific protein and mRNA markers in organoids (100, 119). One can imagine that the entire suite of cell biology tools can be applied to study tuft cells in this setting, such as perturbing tuft cell function through gene knockdowns and engineering fluorescently labeled tuft cells for high-resolution, live imaging. Moreover, tuft cells can be examined in more complex contexts using organoid coculture conditions with other host cell types or microbes. We envision that structural, spatial, temporal, and contextual features of tuft cells can be revealed by next-generation imaging studies across various organoid platforms.

In summary, we have witnessed tremendous growth in the tuft cell field within the past decade, such that these once obscure cells have now become the focus of investigation in a variety of mucosal tissues. Previously defined only by their exaggerated apical tuft, we have now uncovered numerous details of tuft cell architecture and have an advanced understanding of tuft cell–specific lineage specification, signaling pathways, and roles in barrier maintenance. Given the established role of tuft cells in various homeostatic and disease processes in the gut and the notion that intestinal epithelial cells are the new frontier for therapeutic targeting, tuft cells are likely to be an attractive target and will undoubtedly continue to be a strong area of research focus in the coming years.
